# Mitochondrial dysfunction promotes microbial composition that negatively impacts on ulcerative colitis development and progression

**DOI:** 10.1038/s41522-023-00443-y

**Published:** 2023-10-07

**Authors:** Ainize Peña-Cearra, Deguang Song, Janire Castelo, Ainhoa Palacios, Jose Luis Lavín, Mikel Azkargorta, Felix Elortza, Miguel Fuertes, Miguel Angel Pascual-Itoiz, Diego Barriales, Itziar Martín-Ruiz, Asier Fullaondo, Ana M. Aransay, Hector Rodríguez, Noah W. Palm, Juan Anguita, Leticia Abecia

**Affiliations:** 1 CIC bioGUNE, Basque Research and Technology Alliance (BRTA), Bizkaia Science and Technology Park Bld 801 A, 48160 Derio, Spain; 2 Department of Genetics, Physical Anthropology and Animal Physiology, Faculty of Science and Technology, University of the Basque Country (UPV/EHU), 48080 Bilbao, Spain; 3 Department of Immunology, Microbiology and Parasitology, Faculty of Medicine and Nursing, University of the Basque Country (UPV/EHU), 48080 Bilbao, Spain; 4 Department of Immunobiology, Yale University School of Medicine, New Haven, 06519 CT USA; 5 Applied Mathematics Department - Bioinformatics Unit, NEIKER-Basque Institute for Agricultural Research and Development, Basque Research and Technology Alliance (BRTA), Parque Científico y Tecnológico de Bizkaia, P812, 48160 Derio, Spain; 6 CIBERehd, ISCIII, 28029 Madrid, Spain; 7 ProteoRed-ISCIII, 28029 Madrid, Spain; 8 Ikerbasque, Basque Foundation for Science, 48009 Bilbao, Spain

**Keywords:** Clinical microbiology, Microbiota

## Abstract

Recent evidence demonstrates potential links between mitochondrial dysfunction and inflammatory bowel diseases (IBD). In addition, bidirectional interactions between the intestinal microbiota and host mitochondria may modulate intestinal inflammation. We observed previously that mice deficient in the mitochondrial protein MCJ (Methylation-controlled J protein) exhibit increased susceptibility to DSS colitis. However, it is unclear whether this phenotype is primarily driven by MCJ^−/−^ associated gut microbiota dysbiosis or by direct effects of MCJ-deficiency. Here, we demonstrate that fecal microbiota transplantation (FMT) from MCJ-deficient into germ-free mice was sufficient to confer increased susceptibility to colitis. Therefore, an FMT experiment by cohousing was designed to alter MCJ-deficient microbiota. The phenotype resulting from complex I deficiency was reverted by FMT. In addition, we determined the protein expression pathways impacted by MCJ deficiency, providing insight into the pathophysiology of IBD. Further, we used magnetic activated cell sorting (MACS) and 16S rRNA gene sequencing to characterize taxa-specific coating of the intestinal microbiota with Immunoglobulin A (IgA-SEQ) in MCJ-deficient mice. We show that high IgA coating of fecal bacteria observed in MCJ-deficient mice play a potential role in disease progression. This study allowed us to identify potential microbial signatures in feces associated with complex I deficiency and disease progression. This research highlights the importance of finding microbial biomarkers, which might serve as predictors, permitting the stratification of ulcerative colitis (UC) patients into distinct clinical entities of the UC spectrum.

## Introduction

Inflammatory bowel diseases (IBD) are a group of chronic inflammatory disorders that affect the small and large intestines. Ulcerative colitis (UC) is one of the two primary forms of IBD in humans. Although the core etiology of IBD is unknown, altered gut microbiota composition, sometimes referred to as dysbiosis, is a common feature of IBD pathogenesis. Dysbiotic microbiota may influence the disease course through its effects on intestinal immunity^1^. There is growing evidence that specific members of the intestinal microbiota may predispose individuals to disease, although the identification of disease-driving bacteria is still a major challenge^2^.

Recent evidence has revealed a bidirectional interaction between microbiota and mitochondria, emerging as a significant area of research in health and disease^3,4^. In a recent study conducted with a cohort of 408 UC patients, active UC correlated with mitochondrial dysfunction^4^. Importantly, they detected 13 genes associated with reduced ATP production and reduced complex I activity. In previous studies, we observed a profound impact of Methylation-controlled J protein (MCJ, encoded by *Dnajc15*) deficiency on microbial composition during the development and progression of colitis^5^. MCJ is a mitochondrial protein that negatively regulates complex I of the electron transport chain, controlling ATP production without affecting proton leakage and reactive oxygen species production^6^. We have reported that MCJ provides protection against acute colitis and its deficiency affects mitochondrial morphology, gut microbiota composition, and bile acid composition contributing to disease severity^5^. An imbalance of the secondary bile acid pool was also observed in IBD patients with gut dysbiosis^7^. In addition, MCJ deficiency was associated with the enrichment of potential disease-driving bacteria. Our data suggests that gut microbial composition in the colon is affected by MCJ and may impact UC development and progression.

Secretory Immunoglobulin A (IgA) plays a central role in the maintenance of a ‘healthy’ intestinal microbiota and serves as first line of defense of the mucosal surface against enteric toxins and pathogen invasion^8,9^. IgA recognizes a particular subset of microorganisms, either pathogens or commensals, blocking their attachment to epithelial cells and minimizing bacterial translocation through immune exclusion. Palm et al. (2014) reported that high IgA coating, as assessed by a technology called IgA-SEQ, can identify potentially colitogenic bacteria from both murine and human intestinal microbiota^10^. Thus, IgA-SEQ might be a potential tool to identify specific members that are associated with enhanced inflammation in mice with perturbed mitochondrial function.

Here, we characterized the effect of complex I mitochondrial dysfunction on gut microbiota composition and susceptibility to colitis, using MCJ-deficient mice. Our aim was to shed light on the role of microbiota-host mitochondria axis in UC and to identify gut microbial signatures linked to perturbed electron transport chain function. For this purpose, we performed three different approaches based on germ-free (GF) microbial colonization, fecal microbial transplant (FMT) and finally IgA-SEQ technology.

## Results

### MCJ-deficient mice microbial composition increased inflammatory profile in colonized germ-free (GF) mice

**Fig. 1:**
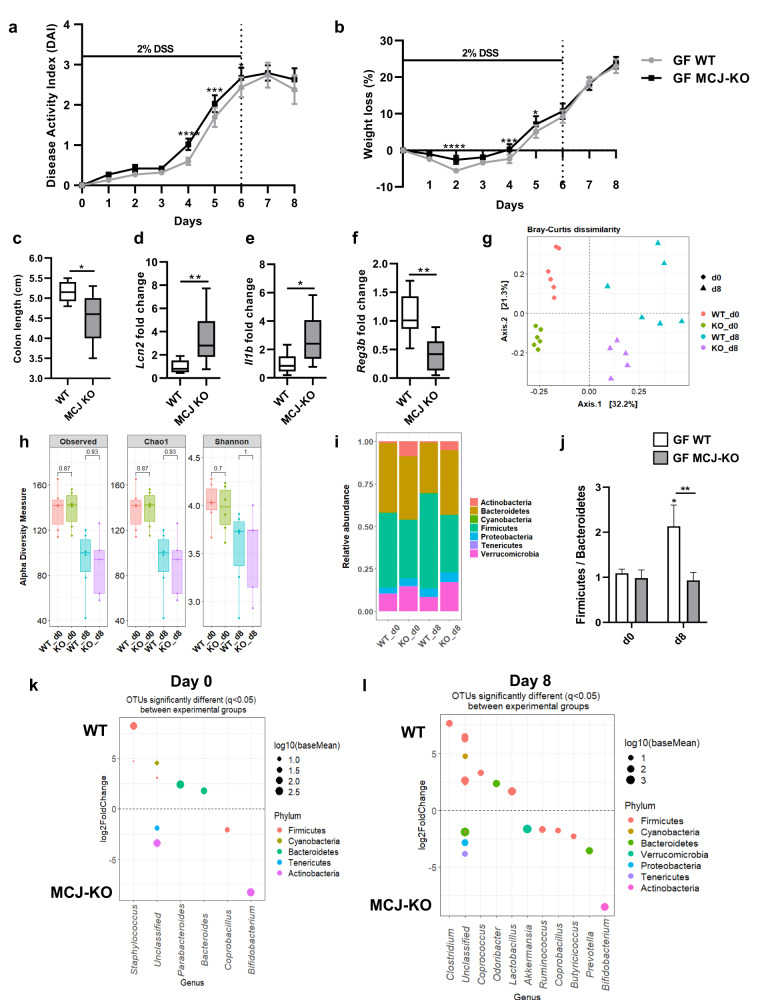
Germ-free mice colonization with WT and MCJ-deficient mice microbial communities. **a** Disease activity index (DAI) and **b** weight loss percentage; data are means ± SEM and analyzed using two-way ANOVA. **c** Colon length (cm). **d**–**f** Gene expression analysis from murine colon tissue of the **d** *Lcn2*, **e** *Il1b* and **f** *Reg3b* genes shown as mean fold change of GF mice colonized with MCJ-deficient microbial consortia compared to GF mice colonized with bacterial community from WT mice. **c**–**f** Data are represented as box and whisker plots of median, quartiles and range with at least 8 mice per group. For statistical analysis, Mann-Whitney *U* test was performed. **g** Principal Coordinates Analysis (PCoA) plot of bacterial beta-diversity based on Bray-Curtis dissimilarities showing experimental groups after bacterial colonization (WT_d0 and KO_d0) and after colitis induction (WT_d8 and KO_d8). **h** Bacterial alpha diversity analysis by means of observed Operational Taxonomic Units (OTUs), Chao1 and Shannon indexes (*P* value < 0.05, Wilcoxon rank-sum test). **i** Stacked bar plots showing the average relative abundance of each group at phylum level. **j** Box plot representation of Firmicutes/Bacteroidetes ratio. Boxes represent mean ± SEM and data were analyzed using two-way ANOVA. “*” above boxes versus control genotype (d8 versus d0), “*” above line versus different genotypes in the same experimental group. **k**, **l** DESeq2 displayed OTUs that were differentially abundant between groups **k** before DSS administration (WT_d0 and KO_d0) and **l** at the end of the experiment (WT_d8 and KO_d8). Each point represents a single OTU colored by phylum and grouped by taxonomic genus. Point´s size reflect the mean abundance of the sequenced data.

To investigate the colitogenic potential of MCJ-deficient mice gut microbial composition, germ-free mice were colonized with WT and MCJ-deficient mouse microbiota. After microbial colonization, no differences in weight were noted. Germ-free mice colonized with MCJ-deficient microbiota were significantly more susceptible to DSS colitis, as measured by DAI (Disease Activity Index) and weight loss, compared to mice colonized with WT microbiota (Fig. 1a, b). Remarkably, stool consistency and occult blood were also aggravated in this group of mice (Supplementary Fig. 1a). At day 8, a marked reduction of colonic length (*P* value = 0.018, Mann-Whitney) was observed in GF mice colonized with MCJ-deficient mouse microbiota (Fig. 1c). The antimicrobial peptide *Lcn2*, a biomarker of intestinal inflammation (Fig. 1d), and the potent inflammatory cytokine *Il1b* (Fig. 1e) were also upregulated in colonic tissues from MCJ-deficient microbiota colonized mice, contrary to the antimicrobial peptide *Reg3b*, which was downregulated (Fig. 1f). However, no differences were detected in the expression of *Tnf*, *Tnfr1* and *Myd88* (Supplementary Fig. 1b). Overall, these data suggest that the dysbiotic microbiota from MCJ-deficient mice exacerbated colitis independent of the direct effects of MCJ-deficiency on the host immune system.

Next, we sought to identify specific groups of bacteria related to UC pathogenesis and altered mitochondrial function that may serve as potential biomarkers of disease progression. Therefore, we sequenced the V4 region of the *16* *S rDNA* gene from fecal content at day 0 (2 weeks after colonization, just before DSS treatment) and at day 8 (two days after DSS treatment stopped). We obtained a mean of 12800 ± 2962 reads per sample and the mean Good´s coverage percentage was 100%. Principal Coordinates Analysis (PCoA) based on Bray-Curtis dissimilarity displayed differences in homeostasis (day 0) between colonized microbial communities based on MCJ contribution (adjusted *P* = 0.006, ANOSIM) (Fig. 1g). DSS treatment and MCJ-deficiency at day 8 were also associated with distinct microbial compositions (adjusted *P* = 0.042, ANOSIM). As reported, alpha diversity indices decreased after DSS administration in both communities (Fig. 1h). Strikingly, bacteria from the Actinobacteria phylum exhibited increased relative abundance in MCJ-deficient microbiota at day 0 compared to WT (*P* value ≤ 0.0001, two-way ANOVA) (8.81% vs 1%) (Fig. 1i). In the DSS treated groups, the relative abundance of the Actinobacteria (*P* value = 0.0044, two-way ANOVA), Bacteroidetes (*P* value = 0.0257, two-way ANOVA) and Verrucomicrobia (*P* value = 0.0075, two-way ANOVA) phyla was significantly higher while Firmicutes (*P* value = 0.0025, two-way ANOVA) were lower, in GF mice colonized with MCJ-deficient microbial community compared to WT. In this regard, the dysbiosis index (Firmicutes/Bacteroidetes ratio) was significantly lower in GF mice colonized with MCJ-deficient mice microbiota compared to those colonized with WT gut microbes (Fig. 1j) as usually observed in IBD. Moreover, DESeq2 identified the OTUs that were differentially abundant at day 0 and day 8 in colon content. Two weeks after colonization and just prior to DSS administration (day 0), *Bifidobacterium* and *Coprobacillus* were increased while *Staphylococcus, Bacteroides and Parabacteroides* decreased in GF mice colonized with MCJ-deficient mouse microbiota (Fig. 1k). Two days after DSS treatment (day 8), MCJ-deficient microbial community showed elevated levels of *Bifidobacterium* but *Butyricicoccus*, *Akkermansia*, *Ruminococcus* and *Prevotella* were also augmented (Fig. 1l). Notably, measured *Ruminococcus* and *Prevotella* changes were confirmed with linear discriminant analysis effect size (LEfSE) (Supplementary Fig. 1c). Conversely, *Clostridium*, *Coprococcus* and *Odoribacter* were lower in GF mice colonized with MCJ-deficient microbiota as compared to mice colonized with WT microbiota.

In conclusion, these results suggest that complex I deficiency affected gut microbial environment, which contributes to the development of the disease when transferred to germ-free mice with non-modified mitochondria.

### Cohousing altered microbial composition and reduced disease severity from MCJ-deficient mice

**Fig. 2:**
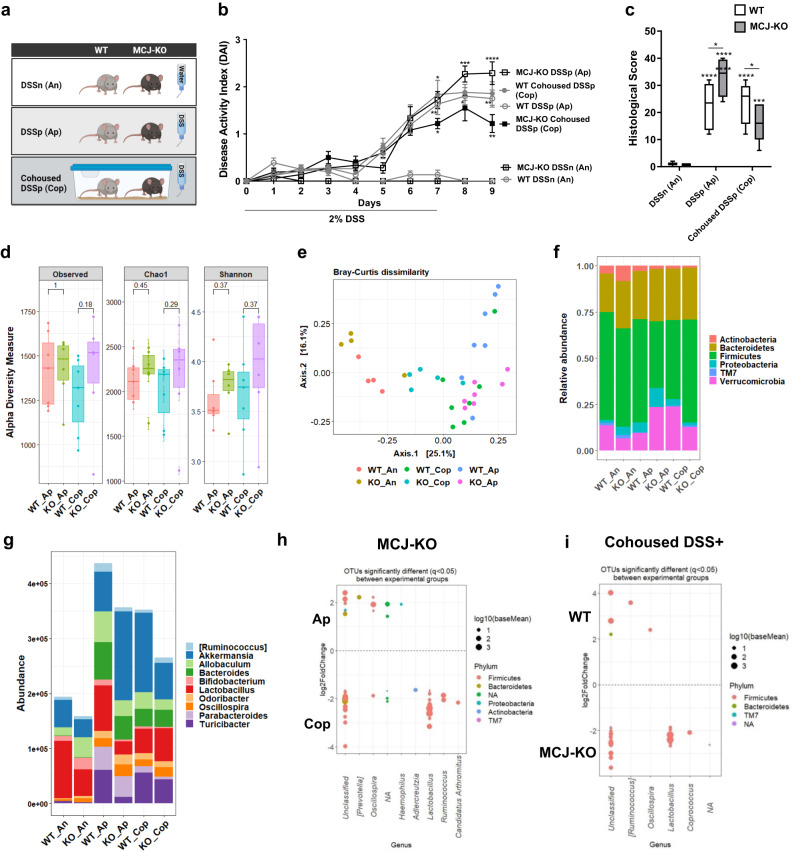
Fecal microbial transplantation via WT and MCJ-deficient animal housing. **a** Schematic representation of the different experimental groups. **b** DAI expressed as means ± SEM and analyzed with a two-way ANOVA statistical test. Statistical differences are represented by asterisks (*) above the line of MCJ-KO DSS-treated alone for MCJ-KO DSS-treated alone and cohoused comparison, below the line of WT DSS positive and alone mice for WT and MCJ-KO DSS-treated alone comparison, and below the line of MCJ-KO DSS-treated and cohoused group, for cohoused DSS positive WT and MCJ-KO mice. **c** Histological score (at least *n* = 9 mice per group in DSS-treated groups). White boxplots indicate WT and grey boxplots MCJ-KO mice. Data were analyzed using two-way ANOVA, where an asterisk “*” above the box shows every DSS positive group versus the control. Differences between genotypes in the same experimental group are presented as a line with an asterisk (*). Significant differences within the same mice genotype between DSS-treated housed alone and cohoused groups are represented with an asterisk (*) inside the box of WT and MCJ-deficient mice that were housed alone (DSS+) **d** Observed OTUs, Chao1 and Shannon alpha diversity indexes (*P* value < 0.05, Wilcoxon rank-sum test). **e** PCoA plot of bacterial beta-diversity based on Bray-Curtis dissimilarities showing distinct grouping between healthy and DSS-treated experimental groups (Statistical test, ANOSIM). **f** Stacked bar chart at phylum level. **g** Relative abundance of the top 10 most abundant genera. **h**, **i** DESeq2 identified differentially abundant OTUs between **h** MCJ-deficient housed alone and cohoused (KO_Ap vs KO_Cop) and **i** cohoused groups (WT_Cop and KO_Cop). WT/KO_DSSn/An: WT and MCJ-KO DSS negative; WT/KO_DSSp/Ap: WT and MCJ-KO housed alone DSS positive; WT/KO _Cohoused DSSp/Cop: WT and MCJ-KO cohoused DSS positive.

To further determine the potential feasibility of microbial manipulation to treat DSS induced damage (aggravated by complex I dysfunction), we cohoused WT and MCJ-deficient mice for 4 weeks (Fig. 2a). First, the disease activity index and histological score parameters were evaluated. MCJ-deficient mice treated with DSS (MCJ-KO DSSp) presented the highest DAI scores at day 8 and 9 (Fig. 2b). However, DAI from MCJ-deficient mice cohoused with WT mice and treated with DSS (MCJ-KO cohoused DSSp) exhibited a significant reduction (*P* value < 0.05, two-way ANOVA). Differences between WT groups were not observed. The histological analysis showed the same tendency, with cohoused and DSS-treated MCJ-deficient mice (MCJ-KO cohoused DSSp) presenting significantly lower histological scores compared to the WT group (*P* value = 0.0269, two-way ANOVA) (Fig. 2c). Moreover, a significant reduction in the histological score was observed exclusively in MCJ-deficient mice treated with DSS following cohousing (Cop) when compared to MCJ-deficient mice housed alone (Ap) (*P* value < 0.0001, two-way ANOVA). Colon length, goblet cells and ROS production were not affected by cohousing (Supplementary Fig. 2a–c). These results indicated that microbes acquired from the WT microbiota during cohousing ameliorate DSS colitis (and dysbiosis) in MCJ-deficient animals.

To determine microbial shifts on WT and MCJ-deficient mice after cohousing and DSS-induced colitis, the V4 region of the 16S rRNA gene from colonic bacterial communities was sequenced. This process generated a mean of 97775 ± 16765 counts per sample and the mean Good´s coverage percentage was 99%. Alpha diversity, determined by the number of observed OTUs, Chao1 and Shannon indexes did not reveal differences between genotypes housed alone (Ap) or together (cohoused, Cop) during intestinal inflammation (Fig. 2d). Principal Coordinates Analysis (PCoA) ordination plot based on Bray-Curtis dissimilarity detected differences between experimental groups (Fig. 2e). NMDS showed a clear cluster between homeostasis (DSSn, An) and DSS-induced colitis groups (DSSp, Ap). Although ANOSIM did not reveal statistical differences between genotypes (WT and MCJ-KO) treated with DSS housed alone or together, WT mice showed statistically different clustering between housed alone and cohoused conditions (*P* value = 0.001, ANOSIM). After one month of cohousing, the data confirmed the reciprocal transmission of gut microbes between both cohoused genotypes impacting significantly in the microbial composition of the WT cohoused group.

Taxonomic differences at phylum level revealed a significant enrichment (*P* value = 0.022, two-way ANOVA) of the phylum Verrucomicrobia in the MCJ-deficient mice housed alone (KO_Ap) compared to WT upon intestinal inflammation. Of note, after cohousing, Verrucomicrobia abundance was significantly increased in WT mice compared to DSS-treated and housed alone WT mice (*P* value = 0.017, two-way ANOVA) (Fig. 2f). Furthermore, the analysis of the top 10 most abundant genera showed substantial shifts between genotypes when the mice were cohoused (Fig. 2g). Potential transmission of *Lactobacillus* and *Turicibacter* genera from WT to MCJ-deficient mice was observed. Subsequently, differential abundance analysis using DESeq2 was performed to test differences in microbial composition between the WT and MCJ-deficient groups housed alone and cohoused. The *Lactobacillus, Ruminococcus, and Adlercreutzia* genera showed higher abundances in the MCJ-deficient cohoused group (KO_Cop) compared to the group housed alone (KO_Ap), indicating that these taxa could have been acquired because of fecal transmission (Fig. 2h). Additionally, *Lactobacillus* displayed a significant enrichment (adjusted *P* < 0.05, DESeq2) in MCJ-KO cohoused mice compared to WT cohoused mice (Fig. 2h). However, the relative abundance of *Prevotella* and *Oscillospira* decreased after cohousing in the MCJ-deficient group (Fig. 2h and Supplementary Fig. 3a). The *Oscillospira* genus augmented in MCJ deficient-mouse microbiota both under healthy and inflammatory conditions (KO_An and KO_Ap) (Supplementary Fig. 3a, b). After cohousing, WT mice (WT_Cop) showed an enriched abundance of the *Oscillospira* genus compared to WT mice housed alone (WT_Ap) and cohoused MCJ-deficient mice (KO_Cop) (Fig. 2i and Supplementary Fig. 3c). Furthermore, taxa from the *Pasteurellaceae* and *Enterobacteriacea*e families were reduced in cohoused mice due to MCJ deficiency (KO_Cop) compared to MCJ-deficient mice housed alone (KO_Ap) (Supplementary Fig. 3d). Pearson´s correlation analysis revealed significant positive associations (*P* value < 0.05) between specific bacterial OTUs abundance and disease severity (DAI), including *Akkermansia muciniphila* (*r* = 0.63), *Parabacteroides distasonis* (*r* = 0.66)*, Bacteroides acidifaciens* (r = 0.63)*, Turicibacter* (*r* = 0.49), *Enterobacteriacea* (*r* = 0.45) and *Prevotella* (*r* = 0.44). On the other hand, the DAI correlated negatively with the S24.7 family (*r* = −0.64), *Lactobacillus* (*r* = −0.63), *Adlercreutzia* (*r* = −0.63) and *Bifidobacterium* (*r* = −0.60) among others. In order to identify whether the treatment with the probiotic bacterium, *Lactobacillus reuteri*, could be effective as an amelioration treatment, we supplemented MCJ-deficient mice with the bacterium and determined pathology upon DSS-induction. Our results showed that the administration of this species (KO Lr + ) did not result in disease improvement in MCJ deficient mice as showed DAI score, weight loss percentage and colonic length (Supplementary Fig. 2e, f).

Collectively, our data show significant microbial shifts after cohousing MCJ deficient and WT mice for one month prior to disease induction. These data also suggest that the effect of these changes on the pathology may be dependent on multiple species that are either elevated or reduced in cohoused MCJ-deficient mice.

### MCJ-deficient mouse microbiota influences immune cell infiltration and regulates the inflammatory response

**Fig. 3:**
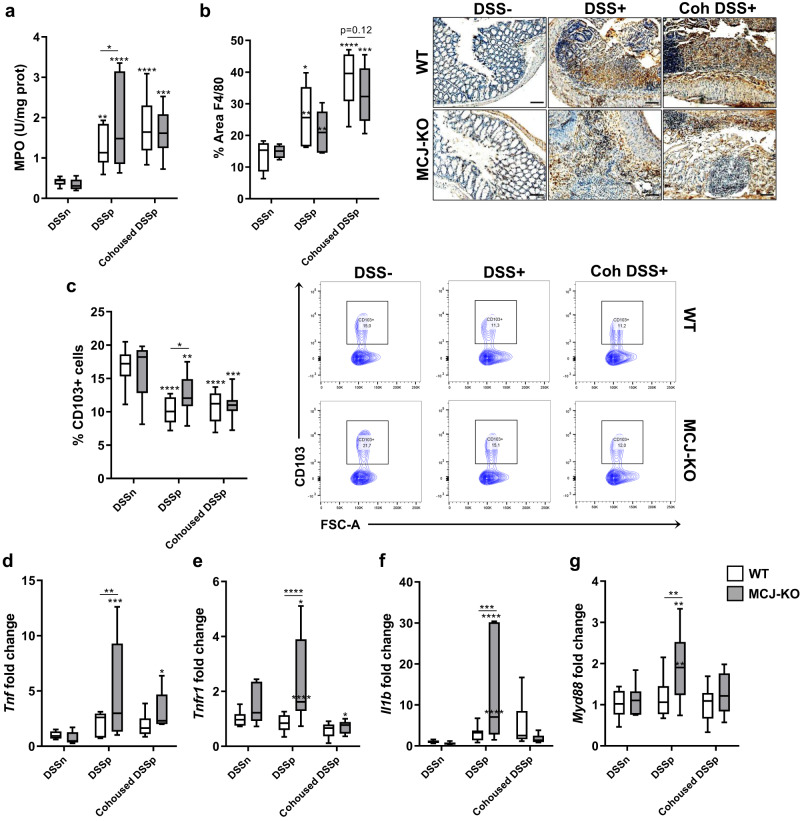
Evaluation of cohousing impact during UC. **a** Myeloperoxidase activity (U/mg prot). **b** Quantification of macrophage area (%) in colon tissue by immunohistochemistry (Anti-F4/80 antibody) and representative images (scale bar, 100μm). **c** Percentage of CD103^+^ dendritic cells from total immune cells within the mesenteric lymph nodes (*n* = 13 within cohoused groups) and representative flow cytometric plots of CD103^+^ population from all experimental groups. **d**–**g** Gene expression analysis from murine colon tissue of **d** *Tnf*, **e** *Tnfr1*, **f** *Il1b* and **g** *Myd88*. **a**–**g** White boxplots indicate WT and grey boxplots MCJ-KO mice. Data are represented as box and whisker plots of median, quartiles and range with at least 8 mice per group (in DSS-treated groups), and at least 7 mice per group (in cohousing groups). For statistical analysis, two-way ANOVA was used as applicable; *P value < 0.05, **p < 0.01, ***p < 0.001, ****p < 0.0001. An asterisk “*” upside the box shows significant differences versus the control (DSS−). Significant differences within the same mice genotype between DSS-treated housed alone and cohoused groups are represented with an asterisk (*) inside the box of WT and MCJ-deficient mice that were housed alone (DSS+). Differences between genotypes in the same experimental group are presented as a line with asterisks (*).

Immune cell infiltration was tested by measuring monocytes, neutrophils myeloperoxidase secretion, and quantifying macrophages and dendritic cells as part of the UC histological features. As expected, MPO levels increased with DSS treatment. Of note, the significant differences (*P* value < 0.05, two-way ANOVA) observed between WT and MCJ KO DSS treated groups, disappeared after 4 weeks of cohousing (Fig. 3a). Infiltration of macrophages in the colon tissue was higher in DSS-treated mice although no differences were observed between genotypes in cohoused and alone groups (Fig. 3b). The percentage of dendritic cells in mesenteric lymph nodes (MLN) decreased upon intestinal inflammation, suggesting the mobilization of these cells to the lamina propria (Fig. 3c). Furthermore, the high percentage of dendritic cells observed in MCJ-deficient mice treated with DSS compared to the WT genotype was not found in cohoused groups. These results indicate that dysbiosis in MCJ-deficient mice may modulate immune cell infiltration with potential implications in intestinal inflammation exacerbation.

Next, we studied colonic gene expression to examine whether specific microbial compositions affect the inflammatory output. *Tnf* expression was increased in MCJ-deficient mice treated with DSS compared to DSS-treated, WT mice. However, this difference was reduced after cohousing, suggesting that the MCJ-deficient microbial community is linked to TNF production and can be modified after one month of cohousing (Fig. 3d). Expression of TNF receptor 1 *Tnfr1*, the proinflammatory cytokine *Il1β* and the adaptor protein *Myd88* were also increased during experimental colitis in MCJ-deficient mice. However, this effect was eliminated in cohoused mice (Fig. 3e–g). Furthermore, the same effect was observed with the antimicrobial peptide *Reg3b* (Supplementary Fig. 2d). Overall, these results suggest that the transmission of microorganisms from WT to MCJ-deficient mice ameliorates intestinal inflammation.

### Interaction between bacterial community and colonic protein levels during cohousing

**Fig. 4:**
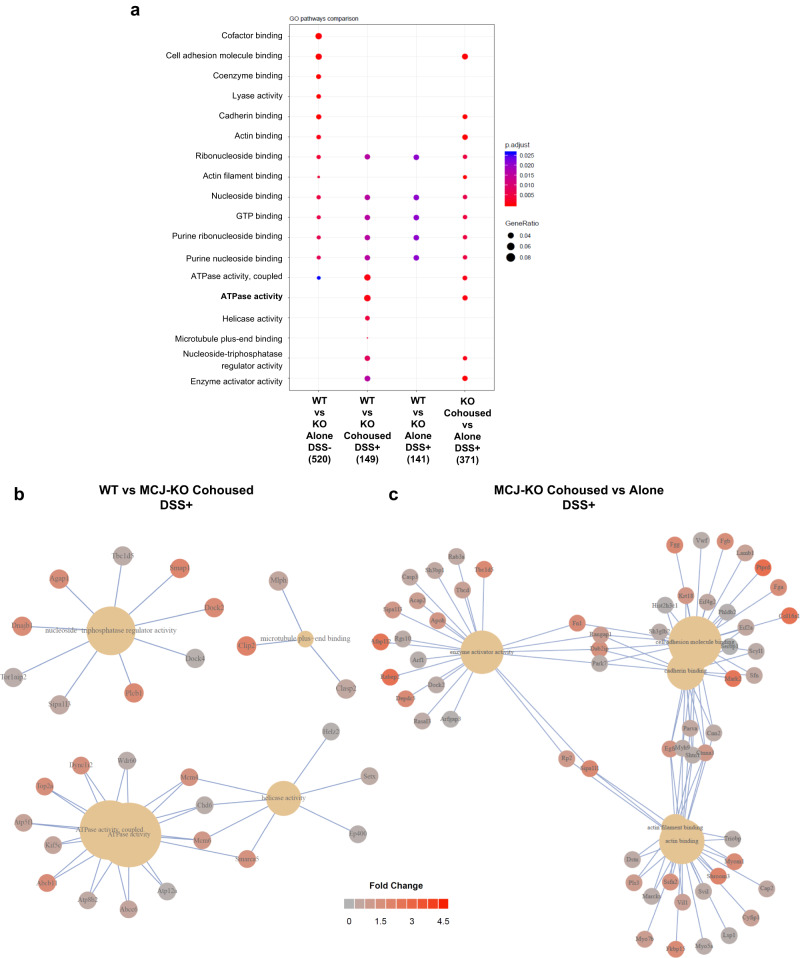
Differential proteome analysis of cohoused mice during UC. **a** GO pathway enrichment analysis. The dot plot shows the up-regulated proteins linked to molecular function (MF) GO pathways (FDR < 0.05) in the different comparisons. Dot size reflects gene count enrichment in the pathway, and dot color displays pathway enrichment significance (adjusted *P*), being red color the most significant. **b**, **c** Proteins implicated in the most significant GO pathways (Molecular function) **b** cohoused (WT vs MCJ-KO) and **c** MCJ defi**c**ient (Cohoused vs Alone) mice with colitis.

To delve into the impact that microbiota composition may have in the gut, we conducted a comprehensive proteomic analysis of the mouse colonic tissue. We identified and quantified a total of 4695 proteins. 153 proteins were differentially (*P* value < 0.05; Student´s *t*-test) expressed between WT and MCJ-deficient mice at steady state (no DSS treatment), 140 between separately housed DSS-treated groups, and 158 between cohoused DSS-treated groups. Functional annotation enrichment analysis revealed several pathways that were significantly different between WT and MCJ-deficient mice (Fig. 4a). In homeostasis, proteins related to molecular function (MF) gene ontology (GO) pathways such as cofactor, nucleoside and actin-binding, and coupled ATPase activity were upregulated in the absence of MCJ. Notably, some proteins associated with MF pathways were enriched in MCJ-deficient mice compared to WT mice only when mice were cohoused, such as ATPase, helicase and enzyme activator activity, which demonstrates the potential of the microbiota to regulate the colonic proteome. Strikingly, separately housed and cohoused MCJ-deficient DSS-treated mice exhibited significant (adjusted *P* < 0.05) shifts in their proteomes including ATPase activity, cell adhesion molecular binding and enzyme activator activity. Microbiota transmission during cohousing also eliminated some differences between separately housed versus cohoused MCJ-deficient mice, such as proteins involved in cell adhesion (Fig. 4a). Furthermore, acquisition of the WT microbiota by MCJ-deficient mice during cohousing affected numerous metabolic processes and the establishment of cell polarity (Supplementary Fig. 4a). To decipher the biological complexity of colon proteomes, we assembled a protein interaction network of the upregulated proteins and pathways linked to MF GO in the cohoused DSS-treated MCJ-deficient mice compared to cohoused WT mice (Fig. 4b). Remarkably, numerous proteins related to ATPase activity and helicase activity were connected. In the protein network that shows the significantly enriched proteins within the GO molecular function in MCJ-deficient DSS-treated mice housed alone compared to cohoused mice, we found multiple interactions between proteins with enzyme activator activity, actin-binding and cell adhesion molecule binding (Fig. 4c). As expected, we also observed increased oxidative phosphorylation in the absence of MCJ, both in healthy and DSS-treated housed alone conditions (Supplementary Fig. 4b). In addition, proteins associated with Cellular Component and KEGG pathways suggested increased mitochondrial respiration (mitochondrial inner membrane, mitochondrial matrix and oxidoreductase complex) and metabolic pathways (carbon, pyruvate and propanoate metabolism, TCA cycle) in MCJ deficient mice compared to WT mice in the absence of DSS (Supplementary Fig. 4b, c). Lastly, in MCJ-deficient mice, cohousing augmented microtubule plus-end binding compared to cohoused WT mice, an essential activity for cell motility, mitosis and intracellular transport (Supplementary Fig. 4b).

**Fig. 5:**
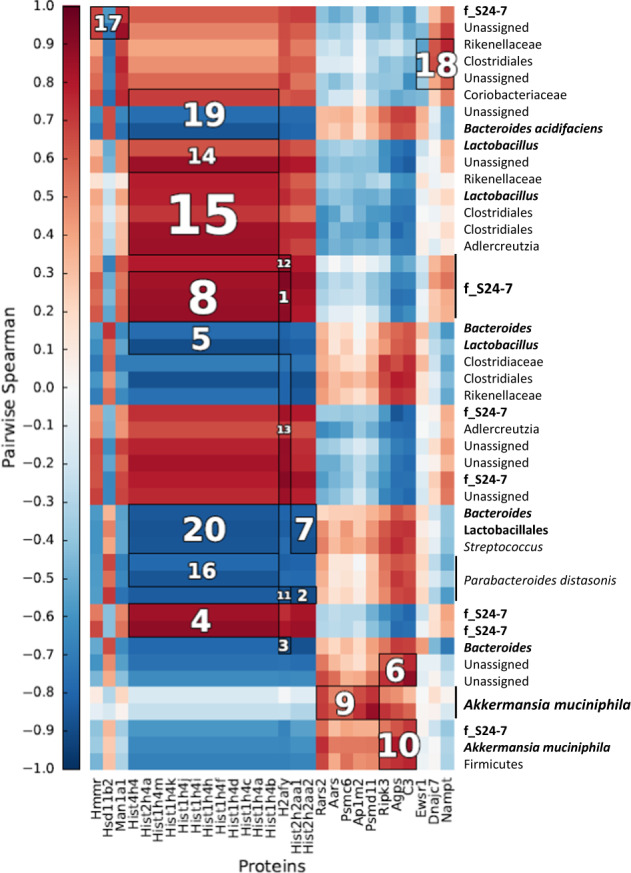
Heatmap of Spearman’s rank correlation coefficients. Associations were determined between bacterial OTUs from WT and MCJ-KO mice treated with DSS and proteins enriched in the colon tissue. To identify significant associations (adjusted *P* < 0.05), Hierarchical All against-All Association (HAIIA) testing was performed. Clusters are ranked according to their significance (adjusted *P*) from 1 to 20 i.e.1 represents the most significant correlation. Red colors illustrate positive correlations and blue colors illustrate negative correlation coefficient.

We performed Spearman correlation analyses to identify associations between proteins significantly different (adjusted *P* < 0.05) between WT and MCJ deficient colitis colon tissue with specific microbial OTUs (Fig. 5). Interestingly, *A. muciniphila*, which was augmented in the MCJ-deficient mice, was strongly associated with AP1M2 protein (adjusted *P* = 0.04), a master regulator of intestinal epithelial cell polarization that is required for maintenance of immune homeostasis. Furthermore, *A. muciniphila* was associated with increased mitochondrial protein translation by tRNA synthetase RARS2 protein (adjusted *P* = 0.04), and is also linked to the appropriate degradation of damaged proteins and cells by PSMC6 and RIP3K proteins. *A. muciniphila* was also related to C3 (adjusted *P* = 0.04), a central component of the complement cascade and part of the innate immune system that plays a key role in defense against pathogens. The S24-7 family, which was generally increased in MCJ-deficient mice, also correlated positively with histone-related proteins, PSMC6, RIP3K and C3 proteins (adjusted *P* = 0.04). Select *Lactobacillus* OTUs also strongly correlated with histone-related proteins (adjusted *P* = 0.04), suggesting that some *Lactobacillus* strains could be involved in the epigenetic reprogramming of immune cells, for example in the process of innate immune memory acquisition. On the contrary, the *Bacteroides* genus and commensal *B. acidifaciens* showed a negative correlation with histone-related proteins (adjusted *P* = 0.04). Collectively, our data indicate that microbiota-host interactions are critical for the development and function of the host immune system in UC.

In summary, transmission of microbiota between distinct genotypes underscores the reciprocal interactions between microbiota and host intestinal tissues, which regulate diverse host functions and responses.

### IgA-SEQ identifies potentially drivers of ulcerative colitis within MCJ-deficiency

**Fig. 6:**
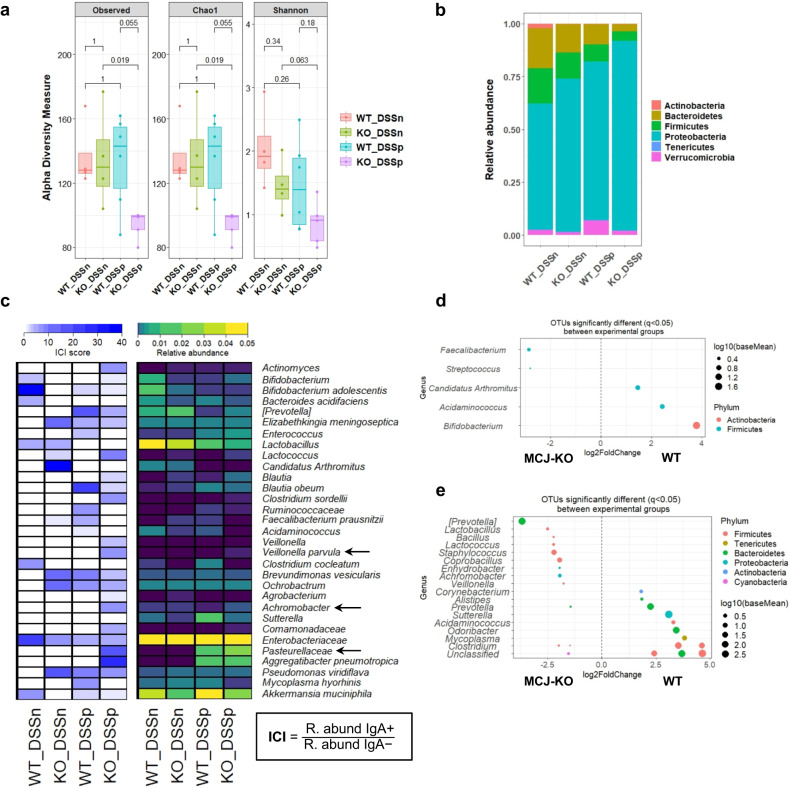
Differential IgA-coating of WT and MCJ-deficient mice intestinal microbiota. **a** Observed OTUs, Chao1 and Shannon alpha diversity indexes of bacteria coated with IgA (IgA + ) (*P* value < 0.05, Wilcoxon rank-sum test) **b** Taxa-bar plot at phylum level of intestinal bacteria coated with IgA in all experimental groups. **c** Heatmap representation of IgA coating index (ICI) score and relative abundances of specific bacterial families, genera and species coated with IgA. To calculate the ICI score the relative abundance of the IgA+ fraction was divided by the IgA- fraction. In the ICI score heatmap, dark blue represents the highest ICI value and in the relative abundance heatmap, yellow color shows the highest relative abundance. **d**, **e** Differentially abundant OTUs identified through DESeq2 testing (adjusted *P* < 0.05) between **d** WT and MCJ-deficient mice gut bacteria coated with IgA in homeostasis (WT_DSSn vs KO_DSSn) and **e** in intestinal inflammation (WT_DSSp vs KO_DSSp).

Previous work in either DSS-induced experimental mice or IBD patients suggested that disease-driving bacteria are highly coated with IgA^10^. Therefore, we sought to determine whether bacterial IgA coating would identify a potential microbial signature associated with complex I deficiency and increased susceptibility to IBD development. We used magnetic activated cell sorting (MACS) and 16S sequencing to characterize taxa-specific coating of the intestinal microbiota with immunoglobulin A (IgA-SEQ). We achieved good taxonomic resolution with a mean read count of 37.502 ± 8.773 sequences per sample and a Good´s coverage percentage of 100%. First, we studied alpha diversity indexes of IgA-coated bacteria including Observed species, Chao1 and Shannon (Fig. 6a). IgA-coated intestinal bacteria from MCJ-deficient mice after DSS treatment exhibited the lowest diversity indexes as compared to IgA-coated bacteria in DSS-treated WT mice (*P* value = 0.055, Wilcoxon rank-sum test) within Observed species and Chao1 indexes. Notably, IgA-coated bacteria in MCJ-deficient mice upon DSS treatment displayed significantly reduced gut microbial diversity compared to those of healthy MCJ-deficient mice (*P* value = 0.019, two-way ANOVA). Proteobacteria were numerically the major phylum coated with IgA in all experimental groups (Fig. 6b). Nonetheless, after induction of colitis, MCJ-deficient IgA-coated taxa displayed significantly higher abundance of this phylum compared to the WT group (*P* value = 0.0458, two-way ANOVA). In homeostatic conditions, IgA coating of Actinobacteria was significantly decreased in MCJ-deficient mice compared to WT mice (*P* value = 0.0352, two-way ANOVA).

High IgA-coating identifies colitogenic bacteria and is associated with increased susceptibility to colitis. Therefore, we compared IgA-coating levels between taxa based on the IgA coating index score (ICI) for each taxon (relative abundance (IgA+)/relative abundance (IgA−)) [10]. In homeostasis, WT mice showed higher levels of IgA-coating and significantly increased relative abundance of *Bifidobacterium adolescentis* (*P* value = 0.0194, two-way ANOVA) (Fig. 6c). On the contrary, the segmented filamentous bacteria (SFB) *Candidatus Arthromitus*, *Elizabethkingia* and *Ochromobactrum* were highly coated in the MCJ-deficient mice whereas they had similar relative abundance in both genotypes, except *C. Arthromitus* that presented higher abundance in WT mice (*P* value < 0.0001, two-way ANOVA) (Fig. 6d). During intestinal inflammation, members of the *Pasteurellaceae* (ICI 18.47 vs 0: *P* value = 0.05, two-way ANOVA) and *Veillonellaceae* (ICI 2.51 vs 0; *P* value = 0.05, two-way ANOVA) families and the genus *Achromobacter* (ICI 6.36 vs 0; *P* value < 0.0001, two-way ANOVA) were more abundant and coated with IgA in MCJ-deficient mice microbiota.

Next, we used DESeq2 to identify statistically different OTUs abundance within IgA-coated bacteria between genotypes. Prior to DSS-induced colitis, OTUs belonging to the genera *Faecalibacterium* (adjusted *P* = 0.018) and *Streptococcus* (adjusted *P* < 0.0001) were enriched in MCJ-deficient mice compared to WT, which instead exhibited increased abundance of *Bifidobacterium* (adjusted *P* = 0.029), *Acidaminococcus* (adjusted *P* = 0.013) and *Candidatus Arthromitus* (adjusted *P* = 0.029) (Fig. 6d). After DSS administration, genera linked to colitogenic members such as *Achromobacter* (adjusted P < 0.0001), *Prevotella* (adjusted *P* < 0.0001)*, Staphylococcus* (adjusted *P* = 0.049) and *Veillonella* (adjusted *P* = 0.015) were augmented in the MCJ-deficient mice, although the putatively beneficial genera *Lactobacillus* (adjusted *P* < 0.0001) and *Lactococcus* (adjusted *P* = 0.0008) were also increased in this group (Fig. 6e). Conversely, members of *Alistipes* (adjusted *P* = 0.018)*, Corynebacterium* (adjusted *P* = 0.018)*, Clostridium* (adjusted *P* = 0.001) *and Sutterella* (adjusted *P* = 0.018) were decreased in MCJ-deficient mice after DSS.

In summary, specific taxa were found to be of higher abundance and IgA-coating in MCJ-deficient mice community and suggest that IgA-SEQ highlights a potential microbial signature linked to complex I deficiency.

## Discussion

Bidirectional mitochondria-microbiota interactions appear to be critical in numerous diseases including obesity, diabetes, intestinal inflammation and cancer^11,12^. We found that the colitis-prone phenotype of MCJ deficient mice was transferable to germ-free mice after microbiota transplantation. Then, mice with altered mitochondrial function improved after cohousing with WT mice, suggesting the acquisition of protective microbial taxa by MCJ-deficient mice during cohousing. Finally, we sought to identify highly IgA-coated bacteria that might contribute to intestinal disease in a mouse model of complex I mitochondrial dysfunction. Notably, microbiome alterations also had dramatic impacts on the host colonic proteome of individuals with altered mitochondria function.

Although most previously described colitogenic bacteria do not induce overt pathology in wild-type mice, they can trigger pathogenic inflammatory responses in genetically or environmentally predisposed animals^10^. This is consistent with our finding that *Ruminococcus* and *Prevotella* are enriched in GF mice colonized with MCJ-deficient microbiota after DSS induction. Strikingly, *R. gnavus* was previously found to be enriched in IBD patients with increased disease activity, and high abundance of this bacterium has been associated with lack of response to anti-TNF treatment^13,14^. *Prevotellaceae* species were reported to exacerbate DSS-induced colitis^15–17^ and to be highly IgA coated in dysbiotic mice^10^. In agreement, *Prevotella* correlated positively with the DAI in our microbial transplantation experiment. The reduced *Oscillospira* and *Prevotella* abundance detected in MCJ-deficient mice after cohousing, suggested that decreased levels of specific microorganisms might be linked to a better prognosis. Low IgA coating of *Oscillospira* has been recently associated with earlier resection and has been considered a potential pathobiont that may exacerbate disease in the absence of a potent IgA response^18^. In agreement with our results, gnotobiotic mice colonized with a community of organisms isolated from IBD patients that included *Oscillospira* were more susceptible to DSS colitis^10^. Furthermore, the high levels of *Il1b, Tnfr1* and *Myd88* expression detected in MCJ-deficient mice housed alone significantly decreased in cohoused MCJ-deficient mice after colitis induction, indicating that specific members of MCJ-deficient microbiota might be responsible for inducing increased pro-inflammatory cytokine production. Based on this data, we confirmed a potential connection between mitochondrial function and microbiota composition by showing that MCJ-deficient mice exhibit a distinct microbial signature opening new venues for the prediction of disease outcome.

After cohousing with WT mice, we found that MCJ-deficient mice acquired many OTUs belonging to the *Lactobacillus* genus. Probiotics, including *Lactobacillus* have been suggested to maintain and induce remission in UC patients, although the efficacy of probiotics in IBD is still inconclusive^19^. Nonetheless, the VSL#3 probiotic mixture (*Bifidobacterium*, *Lactobacillus*, and *Streptococcus*) has been demonstrated to have beneficial effects by inducing remission in active UC patients (56.2% failed to achieve remission with VSL#3 versus 75.2% in patients receiving placebo)^19,20^. Furthermore, multiple OTUs belonging to the *Lactobacillus* genus correlated negatively with the disease activity index. Therefore, the augmented *Lactobacillus* taxa might explain the improvement seen in cohoused MCJ-deficient mice. However, the selected *Lactobacillus* species (*L. reuteri*) did not ameliorate colitis in MCJ-deficient mice suggesting that it might be a marker of disease evolution in this genotype, rather than responsible for the phenotype of mice with mitochondrial dysfunction. Other possibilities are that the phenotype observed could be the result of more complex microbiota changes and not the effect of single species or that the administration period was too sort prior to DSS induction. On the other hand, the positive correlation observed between *A. muciniphila* and the DAI, suggested that *A. muciniphila* might play a therapeutic role to ameliorate the elevated damage produced by DSS-induced colitis^21^. Even though WT mice acquired some members of the MCJ-deficient microbial community, including *Oscillospira* and *R. gnavus*, this did not affect the progression of colitis. In this case, the gut microbial ecosystem is complex and well-established, therefore the presence of dysfunctional mitochondria and the derived environment effect may be needed to develop severe disease, pointing to an essential bidirectional interaction between gut microbiota and mitochondria in mucosal, epithelial and immune cells. However, FMT in GF mice had a stronger effect due to the less effective immune responses and defects in metabolism^22^. Hence, due to the high heterogeneity observed in UC patients, assessment of mitochondrial function in patients has the potential to be an early indicator of the course of the disease, leading to early prognosis, and helping to choose the best therapy in order to obtain a better outcome.

Our understanding of functional disease pathophysiology can be increased by the identification of the proteomic changes in health and disease. These, in turn can have potential implications for improving and promoting personalized medicine. Interestingly, we found that animal cohousing was sufficient to alter the host proteome. Many proteomic pathways affected by cohousing were related to tight junction dynamics that link tissue repair to the innate immune response^23–25^. Collectively, our data suggest that specific members of the gut microbiota, might play an important role in modifying the proteome, including immune responses. These can be associated with complex I, or perhaps more broadly, with mitochondrial dysfunction. For example, the interaction between different *Lactobacilli* species and mitochondrial functions is well-known^26–30^. Overall, our results support the application of proteomics to human IBD to discover biomarkers to predict disease progression or therapeutic responsiveness.

Specific members of the human intestinal microbiota are potentially involved in the development of chronic inflammatory responses. The low diversity of IgA-coated taxa observed in MCJ-deficient mice during intestinal inflammation suggests that IgA coating could be linked to a high affinity, pathogen-specific and T-cell dependent response^10^. Remarkably, we identified *Achromobacter* genus and bacteria belonging to the *Pasteurellaceae* and *Veillonellaceae* families with higher abundance and higher IgA-coating in MCJ-deficient mice after colitis induction. While IgA responses can protect against bacterial driven intestinal inflammation, highly IgA coated bacteria drive inflammatory disease indicating that the host IgA responses to select intestinal bacteria may be insufficient to fully neutralize or clear potentially pathogenic species^10^. Furthermore, IgA coating can also sometimes facilitate bacterial colonization through antibody-enhanced biofilm formation^18,31^. In agreement with our results, a study using a large cohort of pediatric UC patients reported enrichment of the *Enterobacteriaceae*, *Pasteurellaceae*, *Veillonellaceae*, and *Fusobacteriaceae* families in patients with increased inflammation^32^. Furthermore, the *Achromobacter* and *Elizabethkingia* genera have been previously observed in UC patients with active disease^33^. Interestingly, in homeostasis, we found that *Candidatus Arthromitus* and the *Elizabethkingia* genus were highly coated in MCJ-deficient mice. *Candidatus Arthromitus*, also known as segmented filamentous bacterium (SFB), is a potent inducer of the IgA response and Th17 cells and have been suggested to drive intestinal inflammation in UC^34–36^. Of note, SFB appeared to be highly IgA-coated in both colitis-induced, and dysbiosis- and colitis-induced specific pathogen-free mice^10^. Collectively, we conclude that disturbed mitochondrial activity may critically impact microbial composition and host IgA responses, augmenting susceptibility to UC.

In conclusion, our study complements previous findings about the role of the microbiome in UC. Germ-free mice colonization demonstrates that the dysbiotic microbiota from MCJ-deficient mice can confer disease susceptibility. Mitochondrial dysfunction due to MCJ deficiency in UC is associated with increased *Oscillospira* and *Prevotella*, and decreased *Lactobacillus* abundance. However, we show that microbial transplant through cohousing with WT mice improves disease outcomes in MCJ-deficient mice. Therefore, the improvement observed in co-housing experiments in the MCJ-deficient animals is due to the presence of dominant, beneficial species in the gut microbial environment and strongly suggests that the phenotype normally observed in these mice is due to the absence of beneficial species rather than the prevalence of deleterious taxa. Finally, we identify Proteobacteria as the major phylum coated with IgA that are significantly increased in the MCJ deficient groups. Hence, our results show that the microbiota composition is a dominant determinant because the microbiota from MCJ-deficient mice can indeed provoke worse disease by itself (probably due to the invasiveness of MCJ deficient-primed species) even in the absence of the genotype responsible for the selection of this microbiota. Therefore, these results suggest that microbiota-targeted treatment may be a valuable option in patients with similar mitochondrial dysfunction states.

## Methods

### Animals and experimental design

MCJ-deficient and Wild-Type (WT) mice on a C57BL/6 background were used [6] under the approval of Diputación de Bizkaia (Competent Authority) upon favorable review from the Institutional Animal Care and Use Committee at CIC bioGUNE (Spain; permit number CBBA-0615). Mice were maintained under specific pathogen free conditions, applying standard housing conditions (21–23 °C temperature and 12/12-hour light/dark cycles) and fed ad libitum on standard mouse chow (Global diet 2914, Harlam, Madison, USA).

For fecal IgA coating, WT and MCJ-deficient male mice (8–10 week-old mice) were treated with 3% dextran sodium sulfate (DSS) (36–50 kDa; TdB Labs) during 6 days followed by two days of recovery with sterile water. At the end of every experiment, mice were humanely euthanized through administration of CO_2_. Faeces were collected at sacrifice and stored at −80 °C until processing.

For the conventional cohousing experiment, WT and MCJ-deficient female mice between 3–5 weeks of age were mixed during 4 weeks prior to colitis induction. All mice were weaned in our animal facilities. Colitis was induced administering DSS in the drinking water (2%) for 7 days followed by a recovery period of three days with autoclaved water. Feces and tissues were collected at sacrifice and stored at −80 °C until use.

For germ-free mice colonization, GF wild-type C57BL/6 male mice were bred and maintained at Yale School of Medicine and all treatments were made in accordance with Yale Animal Care and Use Committee guidelines. 4–6 week-old germ-free C57BL/6 mice were colonized via oral gavage with 200 µL of WT and MCJ-KO bacterial consortia (50–200 mg feces) from mice of the same sex and age. After 2-4 weeks, 2% of DSS was administered to mice during 6 days followed by two days of water. All gnotobiotic mice were maintained in Techniplast P Isocages and manipulated aseptically for the duration of the experiment. 16S rRNA gene of the V4 region was sequenced from mouse feces at days 0 (before DSS administration) and 8 (2 days after DSS administration) to confirm colonization and microbial composition.

In all experiments, a technician blindly evaluated the disease activity index (DAI) of every mouse on a daily basis. This index is a score based on animal body weight loss, the presence of blood in feces, and stool consistency. The criteria proposed by Camuesco et al., 2004 were used to assign scores^37^.

### Administration of Lactobacillus reuteri to MCJ deficient mice during colitis induction

Colitis was induced in 8–10 week-old MCJ-deficient mice by adding 3% (w/v) DSS to the drinking water for 6 days. *L. reuteri* (1 × 10^9^ CFU per dose) was administered daily from the beginning of the experimental period simultaneously with DSS treatment up to day 8. The *Lactobacillus* species was selected through the analysis of the 16S rRNA in our mouse model. *L. reuteri* was grown in Lactobacilli MRS broth for 20 h supplemented with 0.05% L-cysteine-hydrochloride at 37 °C under anaerobic conditions.

### Determination of ROS in colon tissue sections

Samples were sectioned in a cryostat (8 μm) and incubated with 150 μM of MnTBAP (Santa Cruz Biotechnology) for 1 h at RT. The samples were then washed with PBS and incubated with 1 μM of dihydroethidium (DHE, Sigma-Aldrich) for 30 min at 37 °C. Sections were washed again and mounted with ProLong mounting media containing DAPI (Invitrogen). Photographs were taken with a fluorescence microscope (Axioimager.D1 Zeiss) and analyzed by ImageJ software.

### Histology and immunohistochemistry

Colon tissue was fixed in 10% formalin fixative, dehydrated, embedded in paraffin and cut into 5 μm thick sections. For histopathology, sections were deparaffined, hydrated and stained with hematoxylin. Samples were analyzed by a pathologist blinded to mouse genotype and treatment. The histological score was based on edema, ulceration and infiltration of neutrophils and mononuclear cells in the different layers of the gastrointestinal wall: mucosal epithelium and lamina propia, crypts, submucosa and muscular layer.

For F4/80 immunohistochemistry analysis, tissue sections were deparaffined, hydrated and subjected to antigen retrieval using proteinase K for 15 min. Endogenous enzymes were first blocked with 3% hydrogen peroxide for 15 min and, blocked with goat serum. Sections were incubated with biotin rat anti-mouse F4/80 primary antibody for 2 h at 37 °C (Biolegend Cat#123105; Dilution 1:50), followed by 30 min incubation with ImmPRESS HRP goat anti-rat secondary antibody (Vector Cat#MP-7404-50, Dilution 1:500). Finally, slides were developed with 3,3′-Diaminobenzidine (DAB, Sigma-Aldrich) substrate and counterstained in Mayer’s hematoxylin for 30 seconds. Images were captured with a Zeiss Axioimager A1 microscope and analyzed using the Frida software.

### RNA extraction, cDNA synthesis and gene expression

Colon samples were collected and frozen at −80 °C. The previous day of the RNA extraction, 1 ml of RNAlater solution was added to the samples and stored at −20 °C. Total RNA from colon tissue was extracted using TRIzol (Invitrogen) and Nucleospin RNA kit (Macherey-Nagel) according to the manufacturer’s protocol. M-MLV reverse transcriptase (ThermoFisher Scientific) was used to synthesize cDNA. Real-time PCR (qPCR) was performed on 384 well plates by QuantStudio 6 Flex Real-Time PCR system (Thermo Fisher Scientific) with PerfeCTa SYBR Green SuperMix Low ROX (Quantabio) and amplification was analyzed by QuantStudio Real-Time PCR software v1.3. Primers for *Il1b*, *Lcn2*, *Myd88, Reg3b, Rpl19, Tnf* and *Tnfr1* genes were optimized (see sequences and annealing temperature in Supplementary Table 1). To normalize mRNA expression, the expression of 3 housekeeping genes was measured and *Rpl19* was ranked as the best candidate. The mRNA relative quantification was calculated using the ΔΔCt method^38^, PCR efficiency was always between 90 and 110%.

### Flow cytometry

MLNs were dissected post-mortem and collected in PBS. For lymph node cells preparation, organs were mashed through a 70 μm cell strainer (Falcon), washed and stained with CD103 PE fluorochrome-conjugated antibody (Miltenyi Biotech Cat#130-111-685, Dilution 1:100). Fc receptors were blocked with Anti-mCD16/CD32 (BD Cat# 553142, Dilution 1:400) and only events that appeared as singlets were analyzed. Data was acquired through FACSCanto II flow cytometer and FACSDiva software (BD) and analyzed with FlowJo software. The gating strategy is depicted in Supplementary Fig. 2g.

### Sorting of IgA+ and IgA− bacteria

Feces were homogenized in PBS and duplicates were made for each sample. Presorting samples were centrifuged and froze at −80 °C. Then, homogenates were blocked with rat serum, stained with PE-conjugated Anti-Mouse IgA (eBioscience Cat#12-4204-82, Dilution 1:100) and subsequently, anti-PE microbeads (Miltenyi Biotec Cat#130-048-801, 20 μl of antibody per 10^7^ total cells) were added. A custom-built 96 well magnetic separator (K&J Magnetics) was used for positive selection, followed by negative selection using MACS multi-96 columns (Miltenyi Biotec).

### Fecal DNA extraction and microbiome analysis

Colon content was collected at sacrifice and stored at −80 °C. Fecal DNA from cohousing experiment DNA was isolated using the FavorPrep Stool DNA Isolation Mini kit (Favorgen) following the manufacturer’s instructions. DNA was eluted in nuclease-free Hyclone water, measured spectrophotometrically with NanoDrop (Thermofisher) and stored at −20 °C until use.

Phylogenetic based methods targeting the 16S rRNA gene were used as described in Pascual-Itoiz et al. 2020^5^. Microbial populations present in the colon of experimental mice were characterized by sequencing the 16S rRNA amplicons of the fusion V3-V4 region on an Illumina Inc.’s MiSeq. DNA extracts were used as the template for 16S rRNA gene PCR-based amplification with barcoded primer sets. Data processing was performed using QIIME (v.1.9.0): Quantitative Insights Into Microbial Ecology software package^39^. Sequences were clustered as operational taxonomic units (OTUs) of 97% similarity using UCLUST^40^. OTUS were checked for chimeras using RDP gold data-base and taxonomy was assigned with the Greengenes database (version 4feb2011)^41^. Richness (number of observed species) and alpha and beta diversity metrics (Chao1, Shannon index, and phylogenetic Diversity whole tree) were calculated using the QIIME pipeline. We further performed statistical analyses to detect differences in microbial composition between groups with Vegan and DESeq2^42^ packages for R and the Linear Discriminant Analysis Effect Size (LEfSe) tool^43^. Charts were plotted using several R packages, including phyloseq, ggplot2, ggpubr, reshape2 and qplots among others.

Fecal germ-free mouse DNA samples were extracted with DNeasy PowerSoil Kit following the manufacturer’s instructions. Samples were eluted in 50ul of nuclease-free Hyclone water and measured spectrophotometrically with NanoDrop. The V4 region of 16S rRNA gene of bacteria genomes was sequenced on Illumina MiSeq using barcoded primers. Data was processed with QIIME2^44^ and microbial diversities and charts were obtained with R phyloseq, ggplot2 and vegan packages. The significant fold changes of OTU’s were obtained with DESeq2.

In IgA-SEQ experiment, presorting, IgA positive and IgA negative samples DNA were isolated using MagAttract Microbial DNA isolation Kit (Qiagen). Samples were lysed by adding glass beads together with the lysis solution and bead beating for 5 minutes. Supernatants were transferred to a new plate and DNA was extracted following the manufacturer’s protocol. For microbial sequencing and analysis, the pipeline used with germ-free mouse colonization fecal DNA samples was followed. IgA coating index (ICI) for each individual bacterial taxon was determined dividing the relative abundance of IgA coated bacteria (IgA+) by the relative abundance of non-coated IgA bacteria (IgA−)^10^. Since we tested each sample in duplicate, we used the mean of the duplicates for the analysis.

### Proteomic analysis

Colon samples from the cohousing experiment were digested following the SP3 protocol described by Hughes et al., (2019) with minor modifications^45^. Trypsin was added to a trypsin:protein ratio of 1:10, and the mixture was incubated 2 h at 37 °C. The resulting peptides were dried out in a RVC2 25 speedvac concentrator (Christ), and resuspended in 0.1% formic acid.

Samples were analyzed in a hybrid trapped ion mobility spectrometry—quadrupole time of flight mass spectrometer (timsTOF Pro with PASEF, Bruker Daltonics) coupled online to a nanoElute liquid chromatograph (Bruker). This mass spectrometer takes advantage of a scanning mode termed parallel accumulation—serial fragmentation (PASEF), which multiplies the sequencing speed without any loss in sensitivity^46^ and has been proven to provide outstanding analytical speed and sensibility for proteomics analyses^47^. Samples (200 ng) were directly loaded in a 15 cm Bruker nanelute FIFTEEN C18 analytical column (Bruker) and resolved at 400 nl/min with a 30 min gradient. The column was heated to 50 °C using an oven. Protein identification and quantification was determined using the PEAKS software using default settings. Searches were carried out against a database consisting of human protein entries (Uniprot/Swissprot), with precursor and fragment tolerances of 20 ppm and 0.05 Da. Only proteins identified with at least two peptides at FDR < 1% were considered for further analysis. Data was loaded onto Perseus platform 24^48^ and further processed (log2 transformation, imputation).

GO enrichment was tested using the ClusterProfiler bioconductor package^49^ and comparative graphics were obtained via the dot plot function.

### Pearson correlation

Pearson´s correlation analysis to determine the association between the bacterial OTUs and the DAI of each mouse at the end of the cohousing experiment was performed with the Hmisc R package.

HAIIA (Hierarchical All-against-All significance testing) tool was used to identify significant associations between proteins that were statistically different according to WT and MCJ-deficient genotypes upon intestinal inflammation and all microbial OTU´s (https://huttenhower.sph.harvard.edu/halla).

### Statistical analysis

Statistical analyses were performed using GraphPad software. Results were graphed as line graphs of the mean values and errors, and as box and whisker plots with median, quartiles and range. In the GF experiment, a two-way analysis of variance (ANOVA) was conducted for DAI and weight loss values, and the non-parametric Mann–Whitney *U* test was used for colonic length and qPCR results. In the cohousing experiment, the significance between experimental groups was assessed using the two-way analysis of variance (ANOVA).

### Reporting summary

Further information on research design is available in the Nature Research Reporting Summary linked to this article.

### Supplementary information

Supplementary information

reporting summary

## Data Availability

Raw sequences used for metagenomics analysis were uploaded to the European Nucleotide Archive (ENA www.ebi.ac.uk/ena) under project number PRJEB43545 for the IgA experiment, PRJEB43544 for the Germ-Free mice experiment and PRJEB43553 for the cohousing experiment.
