# Supplementary material for: Mitochondrial dysfunction promotes microbial composition that negatively impacts on ulcerative colitis development and progression
Source: NPJ Biofilms Microbiomes. 2023 Oct 7;9:74. doi: 10.1038/s41522-023-00443-y (PMC10560208; doi:10.1038/s41522-023-00443-y)
Supplement: Supplementary file 1 — Supplementary information [file 41522_2023_443_MOESM1_ESM.pdf]

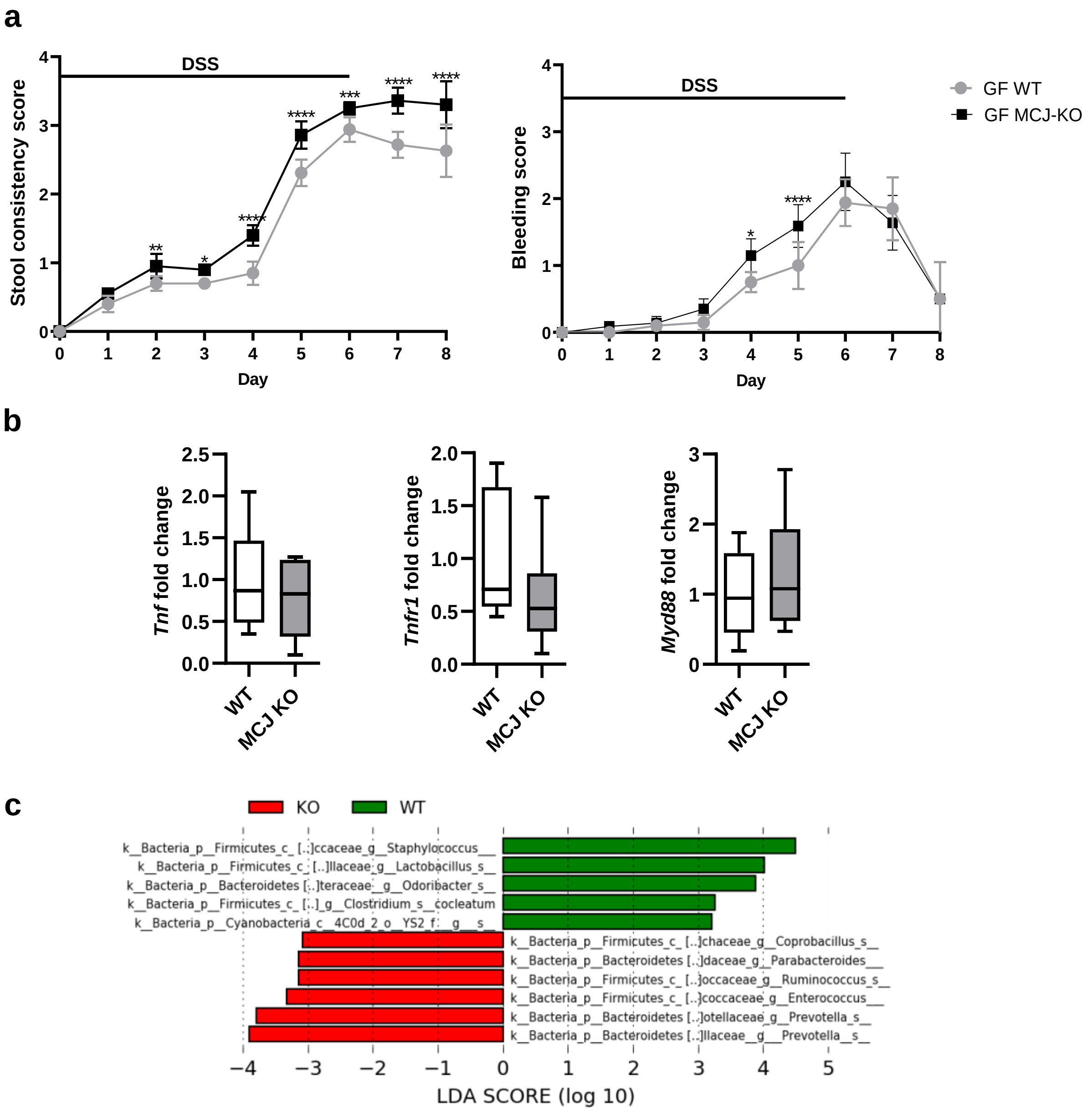

Supplementary Figure 1. Complementary results from the GF mice experiment. a Stool consistency and bleeding score during the experimental period; data are means ± SEM and for statistical analysis, two-way ANOVA was used. **b** Gene expression level of Tnf, Tnfr1 and Myd88 in colon tissue in a GF colitis model. Data is represented as box and whisker plots of median, quartiles and range with at least 8 mice per group and analyzed using Mann-Whitney U test. **c** Histogram of linear discriminant analysis (LDA) effect size (LEfSe) representing statistically differential abundance between GF mice colonized with WT (shown in green) and MCJ-deficient mice (shown in red) microbial communities after colitis induction.

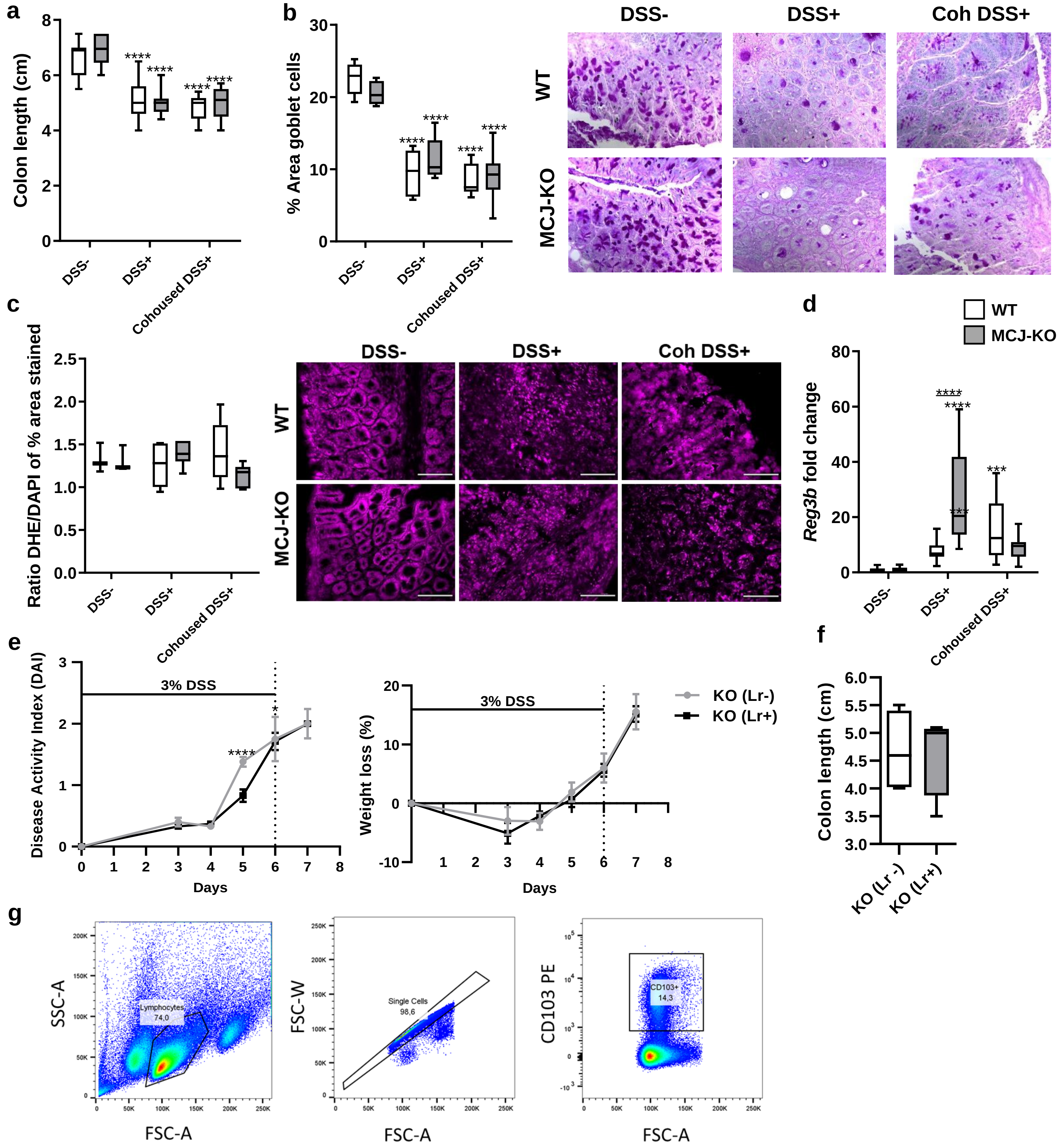

Supplementary Figure 2. Additional data of the cohousing experiment. **a** Colonic length (cm). **b** Goblet cells: % of positive cells stained with PAS (Periodic Acid-Schiff) and representative images of the staining. **c** Reactive oxygen species (ROS) measured by dihydroethidium (DHE) staining in colon sections. Scale bar, 100μm. **d** Gene expression level of *Reg3b* in colon tissue in a DSS-induced colitis model. a-d White boxplots indicate WT and grey boxplots MCJ-KO mice. Data are represented as box and whisker plots of median, quartiles and range with at least 8 mice per group (in DSS-treated groups). Data were analyzed using two-way ANOVA, where an asterisk "*" upside the box shows significant differences versus the control (DSS-). Significant differences within the same genotype between DSS-treated housed alone and cohoused groups are represented with an asterisk (*) inside the box of WT and MCJ-deficient mice that were housed alone (DSS+). Differences between genotypes in the same experimental group are presented as a line with an asterisk (*). **e**, **f** Oral administration of *Lactobacillus reuteri* in MCJ-deficient mice during DSS-induced colitis. **e** Disease activity index (DAI) and weight loss percentage expressed as means ± SEM. **f** Colonic length (cm). **e**, **f** Grey lines and white boxplots indicate MCJ-deficient mice without *L. reuteri* administration, and black lines and grey boxplots MCJ-deficient mice *L. reuteri* orally administrated. For statistical analysis, two-way ANOVA and the non-parametric Mann-Whitney t-test were used. **g** Gating strategy to compare CD103+ cells from the mesenteric lymph nodes of all experimental groups.

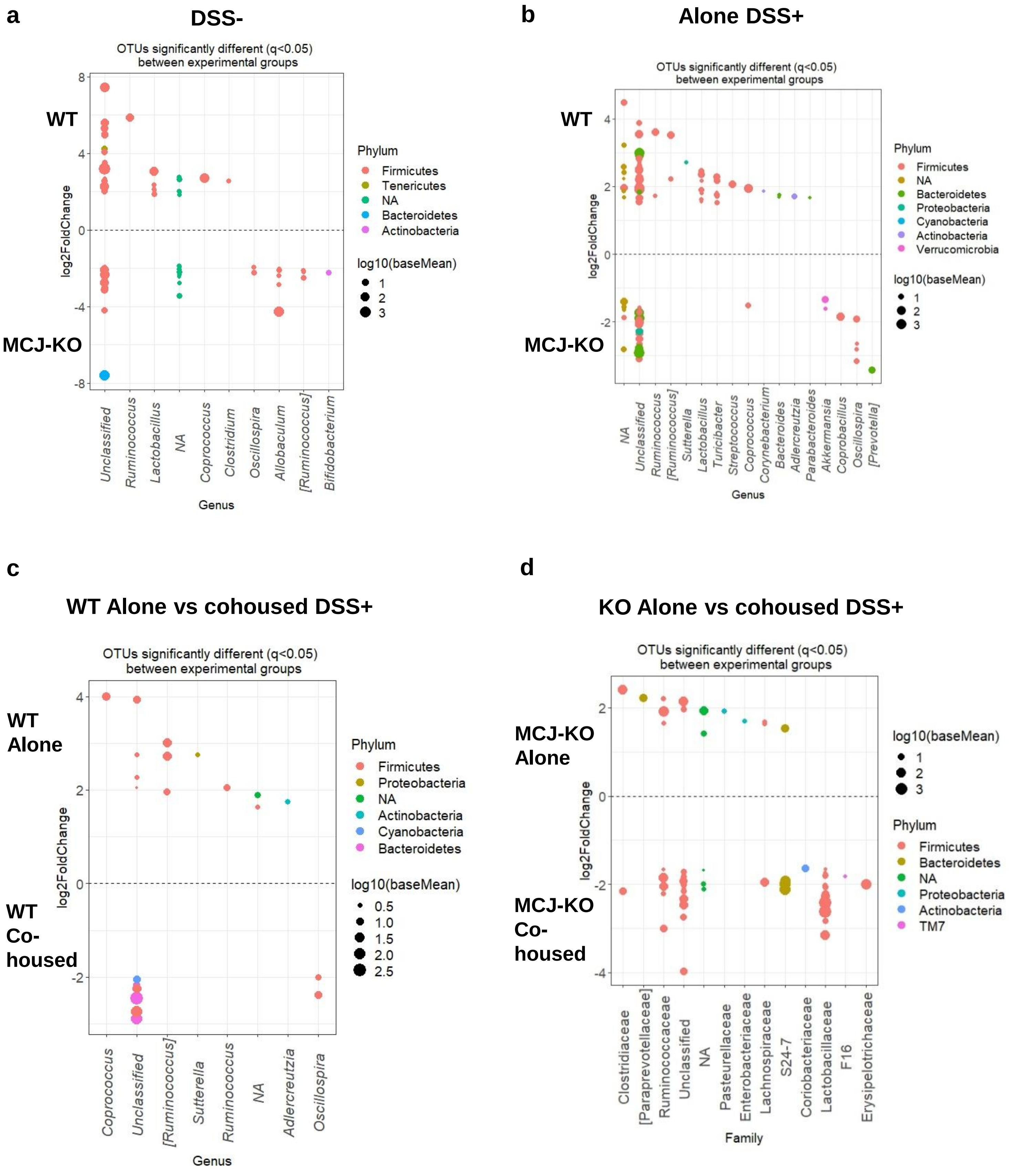

Supplementary Figure 3. Composition of host microbiome at the genus level. Differential representation of OTUs (adjusted P < 0.05, DESeq2) at genus level between **a** healthy (WT vs MCJ KO) housed alone without DSS treatment (DSSn), **b** housed alone (A) DSS positive (p) (WT vs MCJ KO) and **c** WT housed alone vs WT cohoused (Co) DSS p (WT_Ap vs WT_Cop). **d** Differential abundance of OTUs at family level between MCJ-deficient housed alone and cohoused.

a

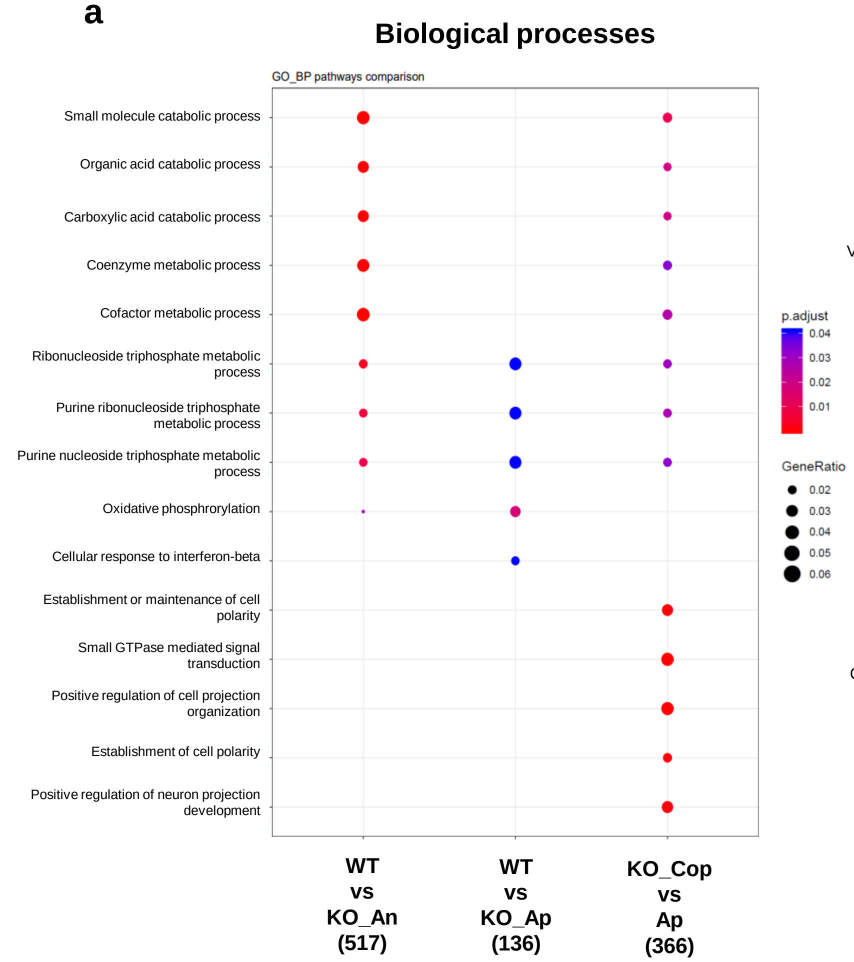

c

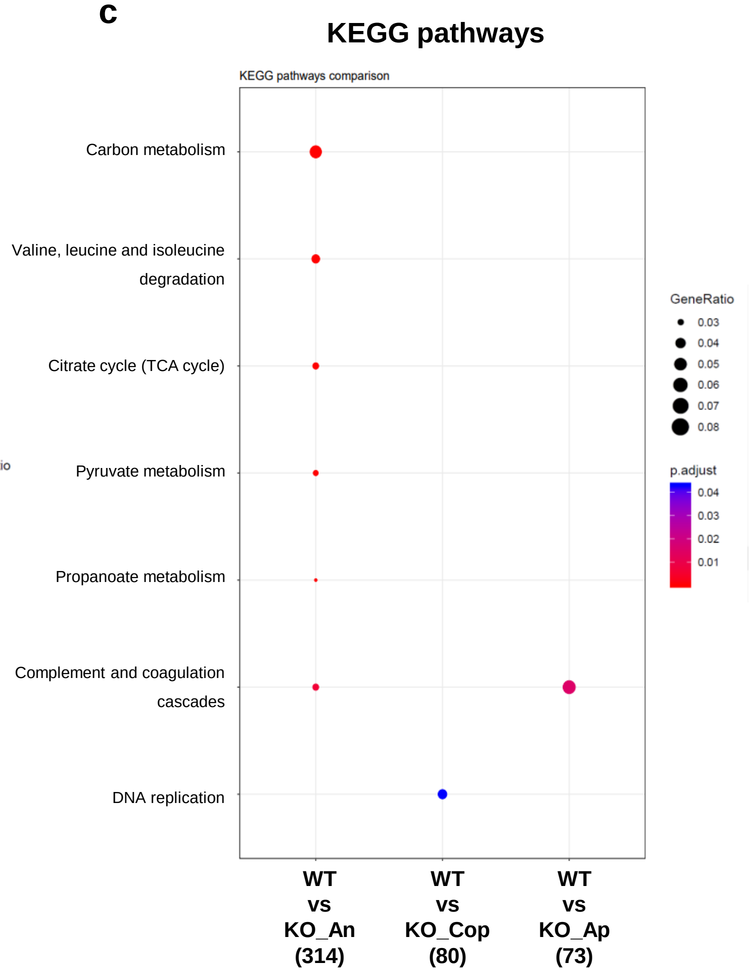

b

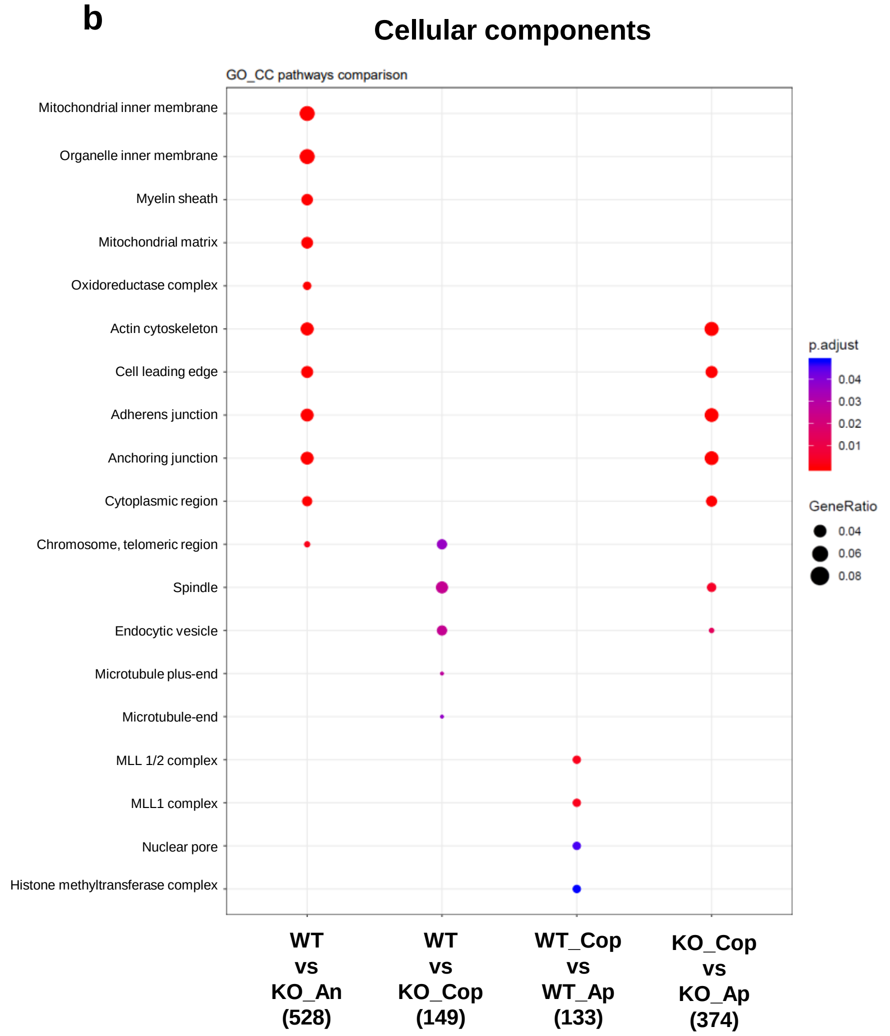

Supplementary Figure 4. The impact of cohousing on the host proteome. Dot plot shows up-regulated proteins associated with the GO terms (FDR <0.05) of **a** biological processes (BP), **b** cellular components (CC) and **c** KEGG pathways identified under the different comparisons. Dot size reflects gene count enrichment, and dot color displays pathway enrichment significance (adjusted P), being red color the most significant. A=housed alone; Co=cohoused; n=DSS negative; p=DSS positive.

Gene	Forward primer sequence<br>Reverse primer sequence	Ta (°C)
Il1b	5´-ACACTCCTTAGTCCTCGGCCA-3´<br>5´-CCATCAGAGGCAAGGAGGAA-3´	60
Lcn2	5´-CAATGTCACCTCCATCCTGGT-3´<br>5´-ACTGGTTGTAGTCCGTGGTG-3´	60
Myd88	5´-CCGCCTATCGCTGTTCTTGA-3´<br>5´-GCCAGGCATCCAACAAACTG-3´	59
Reg3b	5´-TACTGCCTTAGACCGTGCTTTCTG-3´<br>5´-GACATAGGGCAACTTCACCTCACA-3´	60
Rpl19	5´-GACCAAGGAAGCACGAAAGC-3´<br>5´-CAGGCCGCTATGTACAGACA-3´	60
Tnf	5´-AGCCCACGTCGTAGCAAACCAC-3´<br>5´-ATCGGCTGGCACCACTAGTTGGT-3´	60
Tnfr1	5´-GCTGTTGCCCCTGGTTATCT-3´<br>5´-ATGGAGTAGACTTCGGGCCT-3´	60
**Conditions**	Hold cycle 95°C for 2.30', and then 40x (95°C for 15'', 60°C for 1´)	
**Reagents**	PerfeCTa SYBR® Green SuperMix (Quantabio)	
**Instrument**	QuantStudio 6 Flex Real-Time PCR System	
**Expression**	Normalized to *Rpl19* housekeeping gene using the Pfaffl equation and expressed relative to the mean of a relevant control group	

Supplementary table 1. Forward and reverse primer sequences (5′ to 3′) and annealing temperature (Ta) of the mouse primers used for murine qPCR analysis.
